# Genomic Data Support the Revision of Provenance Regions Delimitation for Scots Pine

**DOI:** 10.1111/eva.70038

**Published:** 2024-11-15

**Authors:** Martyna Lasek, Julia Zaborowska, Bartosz Łabiszak, Daniel J. Chmura, Witold Wachowiak

**Affiliations:** ^1^ Department of Genetics and Environmental Interactions Institute of Dendrology, Polish Academy of Sciences Kórnik Poland; ^2^ Department of Plant Ecology and Environmental Protection, Institute of Environmental Biology Adam Mickiewicz University Poznań Poland

**Keywords:** forest tree management, genetic diversity, molecular markers, *Pinus sylvestris* seed zones, SNP genotyping

## Abstract

Scots pine is a crucial component of ecosystems in Europe and Asia and a major utility species that comprises more than 60% of total forest production in Poland. Despite its importance, the genetic relationships between key conservation and the commercial value of Scots pine ecotypes in Poland remain unclear. To address this problem, we analyzed 27 populations (841 trees) of the most valuable Polish Scots pine ecotypes, including the oldest natural stands in all 24 regions of provenance established for the species in the country. By examining maternally inherited mitochondrial markers, nuclear microsatellite loci, and thousands of SNP markers from a genotyping array, we evaluated the genetic structure between and within them. These multilevel genomic data revealed high genetic similarity and a homogeneous structure in most populations, suggesting a common historical origin and admixture of populations after the postglacial recolonization of Central Europe. This research presents novel data on existing genomic resources among local ecotypes defined within strictly managed Polish regions of provenance, challenging their validity. Formal tests of the progeny of seed stands are needed to check whether the diversity in adaptation and quantitative traits still supports the delineation of provenance regions. In parallel, the health status of selected populations and the viability of seeds from these regions should be monitored to detect early‐stage symptoms of their environmental stress. It seems reasonable that periodic shortages of forest reproductive material (FRM) in a given region of provenance could be supplemented with the one from other regions that match their climatic envelope. Together, our results have important implications for the management of native Scots pine stands, particularly elite breeding populations, as they contribute to the discussion of the boundaries of provenance regions and the transfers of FRM that face increasing climate change.

## 1 Introduction

In the face of changing climate, forest tree populations experience increasing environmental pressures, including longer drought periods, intense rainfall, more frequent hurricanes, and outbreaks of pests and diseases (Taeger et al. 2013). As a consequence, they are particularly exposed to increased mortality and will need to adapt or likely change their current distribution ranges following the suitable environmental niche for individual species (Buras and Menzel 2019; Dyderski et al. 2018; Kramer et al. 2010; Saltré et al. 2015). Knowledge of the distribution of genetic variation and relationships between natural populations in the range of species is essential for the development of efficient conservation, management, and breeding strategies for forest trees (Aitken et al. 2008). A better understanding of the genetic relationships between breeding ecotypes representing regions of provenance of the species is particularly important considering the predicted environmental changes that will affect forest productivity and tree mortality rates worldwide (Hall et al. 2007; Phillips et al. 2009).

Scots pine ( *Pinus sylvestris* L.) is a foundation species of various forest ecosystems and one of the most widely distributed and economically important forest trees that is distributed over most of Eurasia. It is a pioneer heliophilic species capable of thriving in various soil types varying in moisture and nutrient content, stretching from coastal regions to altitudes up to 2600 m in mountainous areas (Boratyński 1993; San‐Miguel‐Ayanz et al. 2016). Due to its extensive ecological range, it exhibits considerable phenotypic variability, manifested in various forms, ecotypes, and physiological traits in various climates and forest ecosystems, resulting in more than a hundred morphological varieties described (Carlisle and Brown 1968; Tóth et al. 2017). Phenotypic differences in traits such as bud flush and bud set, height increment, growth rate, frost hardiness, cones and needle morphology, and general breeding quality are extensive within the species, as demonstrated by numerous population studies, for example (Hurme et al. 1997; Andersson Gull and Fedorkov 2004; Hall et al. 2021; Giertych 1993; Barzdajn, Kowalkowski, and Chmura 2016; Oleksyn et al. 2001). In Poland, Scots pine is one of the main forests‐forming and utility species covering more than 5.5 million hectares and represents the largest growing stock (more than 60% of gross wood production) among all other forest tree species.

Earlier studies using various genomic methods provided much data on the demographic history and genetic structure of *P. sylvestris* throughout its wide range of distribution (Bruxaux et al. 2024; Łabiszak and Wachowiak 2023; Żukowska et al. 2023; Kavaliauskas, Danusevičius, and Baliuckas 2022). The studies identified several genetic linages of the species, but found mostly uniform genetic structure in large geographical areas with very little between‐population and high within‐population variation. Some signatures of significant population divergence were observed in isolated areas of old refugial regions (Pyhäjärvi, Salmela, and Savolainen 2008; Naydenov et al. 2007; Dering et al. 2017, 2021). However, it is unclear how this range‐wide pattern translates to genetic relationships of the primary, oldest populations that were used for breeding. Furthermore, genetic data are needed to advance management strategies for stands that have been valued for decades in silviculture and are maintained under strict regime of regions of provenance.

Throughout the years, numerous attempts have been made to determine the genetic value and elucidate the diversity of the most precious pine populations in Poland. Based on morphological variation, growth and productivity traits, but also biochemical studies, many types, ecotypes, or even breeds have been described and valued in forestry due to the unique phenotypic characteristics of a given population (Remlein et al. 2015; Giertych 1997; Staszkiewicz 1993; Przybylski, Matras, and Sułkowska 2015; Hebda and Wachowiak 2019). However, the origin and genetic relationships between those stands could not be well defined based on morphological or anatomical traits including cone or needle biometry as those vary greatly depending, among others, on habitat, latitude, height above sea level, mating processes in the stand, age of the trees and even by location in the crown (Staszkiewicz 1993; Zajączkowska et al. 2020). To evaluate the repeatability of the characteristics of the most famous ecotypes of Scots pine, many international provenance trials have been established over the years to validate their growth performance and survival. The experiments revealed that the populations of species are characterized by a high diversity of phenotypic and quantitative traits (Hurme et al. 1997; Savolainen and Pyhäjärvi 2007; Shutyaev and Giertych 1997; Perry et al. 2016). The results from international IUFRO experiments indicated that Scots pine populations from Poland belong to the most adaptive and well‐performing ones exhibiting a better growth compared to those of other parts of the European range of the species (Giertych 1993, 1991; Giertych and Oleksyn 1992).

According to the regulations of the European Union, each country should determine the provenance regions for each tree species subject to production and marketing of forest reproductive material (FRM). These activities ensure that seed regionalization and seedling transfer prevent the negative effects of uncontrolled movement of reforestation material. Regions of provenances are the areas with uniform ecological conditions where stands show similar phenotypic or genetic characters, taking into account altitudinal boundaries (Council of the European Union, 1999 Council Directive 1999/105/EC of 22 December 1999 on the marketing of FRM). In Poland, there are currently 24 regions of provenance of Scots pine that determine the rules for the use and transfer of FRM (Figure S1). According to this delineation, no outside reproductive material is allowed for use in some of the regions of provenance (assigned with the second digit of their signature different than zero, for example, So11, So21, etc. see Table S1 and Figure S1), and detailed deployment rules are given for the other regions (Regulation of Polish Minister of the Environment of July 29, 2015 on the use of FRM outside the region of origin, Journal Laws of September 8, 2015, item 1328). Typically, the basic material within these provenance regions consists of production seed stands (PSS) and registered seed stands (RSS). The latter are excluded from intensive forest management and are intended exclusively for the production of FRM. Both PSS and RSS can include trees with outstanding phenotypic characteristics (plus trees) dedicated to seed harvesting and the establishment of seed orchards. The rationale behind this delineation was mainly to preserve the gene resources of populations in a given area that are presumably adapted to local environments (Fonder, Matras, and Załęski 2007). However, it is largely unknown to what extent this practice may need some review or makeover. This uncertainty emerges from several factors, such as gene exchange in wind‐pollinated species, the influence of foreign pollen from outside provenance regions, mostly unknown genetic basis of ecotype variation including plasticity of some phenotypic traits, and most importantly, increasing pressure of environmental changes that will shift adaptive optima of local populations. Furthermore, due to the lack of suitable molecular tools, the genetic relationships between the populations of Scots pine of key importance in Poland remain unclear.

The following research provides multilevel genomic characteristics and an assessment of mutual genetic relationships between Polish Scots pine populations, representing all regions of provenance and some of the oldest stands (average 165 years) that were recognized as valuable ecotypes of superior phenotype or breeding value and were protected to preserve putatively local gene pool of the species. Those most valuable stands of this species in Poland are managed under strict regime of the regions of provenance. A comparative analysis of those old populations was performed on a large set of genetic markers for the species, including mitochondrial DNA markers and polymorphisms in thousands of genomic regions analyzed with the application of microsatellite markers and SNP genotyping technology. The research provides new data on existing genomic resources and genetic relationships between defined ecotypes. As breeding populations and regions of provenance of Scots pine are the foundations of modern forest management in many European countries, our study provides important information on existing genomic resources of species to support management decisions in light of rising tree mortality rates and loss of its adaptive optima. Therefore, the results contribute not only to genomic diversity studies of keystone forest tree species but also to ongoing discussion on their breeding and management strategies following environmental changes that affect many populations throughout the range of species distribution.

## 2 Materials and Methods

### 2.1 Plant Material and DNA Extraction

**FIGURE 1:**
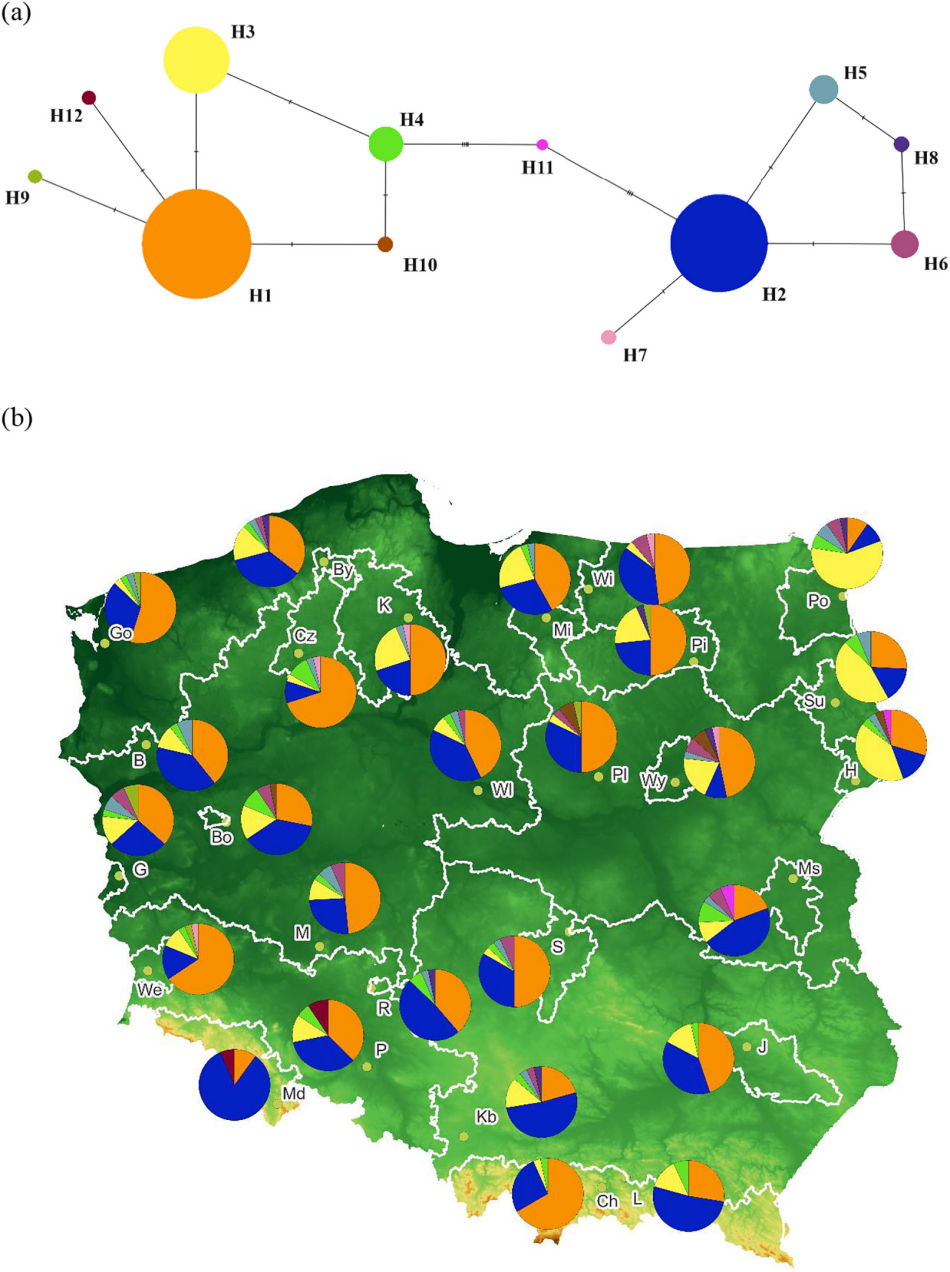
Network of twelve major *mt*DNA haplotypes detected (a) and their spatial distribution in Polish Scots pine populations (b). Boundaries of the analyzed provenance regions are marked with white line based on the data from Polish Forest Seed Office https://www.bnl.gov.pl/. Population abbreviations are presented in Table 1.

Scots pine needles were collected from 27 native populations in Poland representing all regions of provenance (So) for the species in the country (Figure S1). 841 trees included in this study (28–33 from each location) come from strictly managed RSSs or conservation stands and represent the oldest and most valuable breeding material of the species in the country (Figure 1 and Table S1). Several plus trees from the analyzed populations were also included in the study. Genomic DNA was extracted from fresh needles using a Genomic Mini AX Plant kit (A&A Biotechnology, Poland) following the standard manufacturer protocol. The quality of the extracts was evaluated using a BioPhotometer plus (Eppendorf AG, Germany), and the DNA concentration was adjusted to 40 ng/μL.

### 2.2 Mitochondrial DNA ( *mt*DNA) Variation

A set of 12 mitochondrial markers (*mt*DNA) (PR: 5, 7, 15, 19, 20, 21, 24, 25, 29, 30, 31, 32) and NAD1 intron B/C were genotyped following the SnapShot method described in (Szczepański, Łabiszak, and Wachowiak 2023). The multilocus genotypes of all markers and spatial *mt*DNA haplotypes (mitotypes) were visualized using QGIS v.3.36 (Figure 1). The phylogenetic relationship among haplotypes was examined in POPART v.1.7 (Bandelt, Forster, and Röhl 1999). HaplotypeAnalysis v. 1.05 software (Eliades and Eliades 2009) has been used to calculate the number of haplotypes detected in each population (H), the effective number of haplotypes (Ne), the haplotypic richness (Rh) and the haplotype diversity (Hd) at the population level. Linear regression equations were fitted between the four measures of genetic diversity and the latitudes of population origin in R v. 4.0.3 (R Core Team, 2022). Furthermore, the relationships between populations were visualized based on the unweighted pair group method with the arithmetic mean function (UPGMA) in the software R v.4.0.3 and visualized in FigTree v 1.4.4. The phylogeographic structure was then tested using two measures of population differentiation, including G_ST_ based on haplotype frequency (Nei 1973) and N_ST_ based on haplotype similarity (Lynch and Crease 1990) in PermutCpSSR v.2.0 (Pons and Petit 1995). We performed the Mantel test to analyze genetic and geographic distances between populations using GeneAlex (Peakall and Smouse 2006). Finally, to define populations that are geographically uniform and maximally differentiated from each other, we performed SAMOVA 2.0.

### 2.3 Nuclear Microsatellite ( *n*SSR) Variation

16 nuclear microsatellite loci (*n*SSR) described previously (Provan et al. 1998; Elsik et al. 2000; Sebastiani et al. 2012; Chagné et al. 2004) were amplified in three multiplex PCRs (Żukowska et al. 2017) using the Qiagen Multiplex PCR Kit (Qiagen, Germany). The PCR reaction contained 5 μL of Qiagen Multiplex Master Mix, 1 μL of Q‐Solution, 0.2 μL of primer mix, 1,8 μL of water and 2 μL of DNA template. The multiplexes comprised the following markers: (I) psyl18, psyl25, psyl36, psyl42, psyl44, psyl57; (II) ptTX2146, ptTX3025, ptTX4001, ptTX4011, spac11.4; (III) ptTX8446 psyl17, pTctg4363, spac12.5. The PCR products were fluorescently labeled and along with the internal size standard GeneScan 500 LIZ (Thermo Fisher Scientific, USA), were separated on the Applied Biosystems 3130xl Genetic Analyzer (Thermo Fisher Scientific, USA). Allele sizes (length of fragments) were determined using GeneMapper software v. 4.0 (Thermo Fisher Scientific, USA). Subsequently, the lengths of fragments, representing allele sizes, were established utilizing GeneMapperTM software v. 4.0 (Thermo Fisher Scientific, USA). All size varieties were manually verified and altered. FreeNA software (Chapuis and Estoup 2006) was run to verify the frequency of null alleles, using a maximum likelihood method and employing the expectation maximization algorithm. The basic genetic parameters were calculated using GenAlEx v. 6.5 (Peakall and Smouse 2006) including the mean number of alleles (A), the number of private alleles (AP), and observed heterozygosity (H_O_) and expected heterozygosity (H_E_). Deviations from Hardy–Weinberg equilibrium (HWE) in all tested populations were evaluated in GENEPOP v. 4.7.5 (Rousset 2008). To estimate the inbreeding coefficients (FIS) and mean rarefied allelic richness (Ar), we employed FSTAT v. 2.9.4. (Goudet 1995, 2002). We also adjusted the FIS values for the occurrence of null alleles with a Bayesian approach executed in the INEst software v. 2.2 (Chybicki and Burczyk 2009). To assess the level of relatedness across our samples and populations, we calculated pairwise kinship estimates using the Queller‐Goodnight relatedness measure (Queller and Goodnight 1989) with the related package in R (Pew et al. 2015). If one population had individuals that were more related to each other than those in other populations, it could potentially bias some population structure analyses. Both the SSR and SNP datasets (see below) were used for these calculations. The relatedness measure ranges from −1 to 1, where negative values indicate individuals are less related than the average, 0 indicates no relatedness beyond what is expected by chance, and 1 represents clones or selfing individuals. To deduce genetic relationships among populations and individuals, we implemented the principal component analysis (PCA) and principal coordinate analysis (PCoA) using a matrix of pairwise Nei genetic distances. Furthermore, we have evaluated the genetic correlations between populations applying STRUCTURE v. 2.3.4 (Pritchard, Stephens, and Donnelly 2000) with 500,000 iterations conducted after a burn‐in period of 50,000 with 20 independent runs established for the number of genetic groups from K1 to K12. StructureSelector software (Li and Liu 2018) was used to define the optimum K value. Additionally, we have conducted the molecular variation analysis (AMOVA) with 1000 permutations in Arlequin v. 3.5.2.2 (Excoffier and Lischer 2010). Finally, the genetic, environmental, and geographic variables of the analyzed stands were used to assess the genetic structure in our dataset using POPS 1.2 (Jay et al. 2015).

### 2.4 Single‐Nucleotide Polymorphisms (SNPs) Variation

We genotyped 49,829 SNPs that were used for the development of the PiSy50k SNP array (Affymetrix, Thermo Fisher Scientific, USA). Details about the array design and its validation are described in Kastally et al. (2022). Briefly, the array comprises polymorphisms discovered in exom capture, transcriptome, and candidate genes resequencing in Scots pine. Genotyping was performed in 384‐well format on a GeneTitan (Affymetrix, Thermo Fisher Scientific, USA) at Bristol Genomics (UK) after DNA amplification, fragmentation, chip hybridization, single‐base extension through DNA ligation, and signal amplification performed according to the Affymetrix Axiom Assay protocol. Genotype calls were obtained using the Axiom Analysis Suite software as recommended by the manufacturer (Applied Biosystems, USA). The initial set of 43,565 SNPs genotyped with the PiSy50k array was examined and filtered to remove low‐quality samples and markers. A maximum of 5% of the missing loci per sample was chosen as sample quality. Similarly, SNPs were removed if they were successfully genotyped in less than 5% of the samples, were monomorphic or had a minor allele frequency (MAF) below 1%, or if they showed a significant deviation from the Hardy–Weinberg equilibrium (*p* < 0.05) calculated using PLINK v.1.9 (Purcell et al. 2007). Furthermore, the resulting set of clean data was tested for possible linkage between loci, and one of the SNPs in each linked pair was removed to eliminate disequilibrium in the data set. These LD associations were tested and pruned using PLINK v 1.9 based on squared correlations (argument “–*r* ^2^”) and its default value of 0.2. Therefore, most of the analyses were performed using the entire SNP data set and LD‐pruned SNP datasets. The level of genetic variation present between populations was calculated based on the number of private polymorphisms, the average number of nucleotide differences (*K*), the expected and observed heterozygosity, and the coefficient of inbreeding in each population using DnaSP v. 6.12.03 and Arlequin. We examined the distribution of the inbreeding statistic (*F*) across SNP loci within populations as elevated *F* values would indicate higher inbreeding and thus relatedness between samples. Genetic distances between populations were estimated based on the raw numbers of pairwise differences and calculated in Adegenet v.2.1.9 (Jombart 2008). Furthermore, Mantel isolation by distance (IBD), which estimates the correlation between geographic and genetic distances between populations, was carried out in Adegenet using Edwards distances and 999 permutations. PCA and PCoA were performed to discriminate samples and populations. The population structure and genetic relationships between populations were further evaluated on the average number of pairwise differences, F_ST_ statistics, and the analysis of molecular variance (AMOVA) conducted in Arlequin. To properly handle information obtained from genotyped SNPs, we chose to consider unphased data as multilocus data with unknown gametic phase in all the Arlequin analyses. Furthermore, the samples were grouped following the individual‐based Bayesian clustering method, using STRUCTURE v.2.3.4 (Falush, Stephens, and Pritchard 2003). It was launched using Structure_threader v.1.3.0 software (Pina‐Martins et al. 2017), which allows for parallel computation and automated run times. Four parallel runs, starting with different seed values, were carried out across the entire SNP data set with two replicates in each run (“‐R 2”) and other default settings to test how the samples group when split into different numbers of groups of 1 to 30 (“‐K 30”). As the runs of the original STRUCTURE program, performed on the full SNP set as well as on the LD‐pruned set, appeared overwhelmingly time consuming even when Structure_threader was involved, we decided to prepare 4 subsets of 5000 random SNPs taken from each set and check the coherence of the results across these runs. The sample grouping into up to 30 “populations” was tested; all runs started with 50,000 burn‐ins followed by 50,000 MCMC iterations. Mixed and no mixed models were used, with default settings, except that we used correlated allele frequencies with an initial value of α set to 0.04, based on previous inspection. No prior information on the origin of the samples was used, and four replicates (“‐R 4”) were used for each analysis. To recognize the best number of groups similar to the potential population, we analyze four summary statistics based on the logarithmic probability of the data: mean ln*p*(*K*)—averaged over 4 replicates, ln'(*K*), | ln'(*K*)' | and Delta *K* (after (Evanno, Regnaut, and Goudet 2005)).

To identify SNPs with allele frequencies significantly deviating from neutrality across the regions of provenances studied and to assess the impact of these SNPs on population structure and differentiation, we performed an outlier detection analysis using two complementary approaches: an F_ST_ outlier scan with OutFLANK v0.2 (Whitlock and Lotterhos 2015) and a population structure‐based detection method implemented in the pcadapt v4.3.3 R package (Privé et al. 2020). The use of both methods helps address the inherent challenges of multiple statistical tests, which can increase the probability of detecting and reporting false positive outliers. We set thresholds for the false discovery rate (FDR) at 0.05 and 0.1 using the package R qvalue (Storey, Bass, and Dabney 2022). We visualized the identified outliers for both methods using Manhattan plots and Venn diagrams drawn using ggplot2 and (Wickham 2011, 2016). Since SNPs under local adaptation are likely to be detected by both methods, and recognizing that some outliers may be false positives despite FDR control, we considered only the robust set of loci concordant between both methods as potential adaptive variants. Next, to assess the effects of outlier SNPs on population structure, we performed a PCA on three sets of SNPs: the full set of SNPs, the set excluding outliers, and the set including only outliers. We wanted to explore whether outlier SNPs influence the overall genetic structure among populations. By comparing the result of PCA without outliers vs. with only outliers, we could detect whether the population structure is driven mainly by neutral variation or if it is potentially confounded by loci under selection. Additionally, we performed an admixture analysis using sparse nonnegative matrix factorization in the LEA package using only outliers to formally explore the population structure (Frichot and François 2015). LEA analysis allowed us to identify population clusters and estimate ancestry proportions, providing additional information on the genetic structure shaped by outlier SNPs. We tested ancestral clusters ranging from *K* = 1 to *K* = 30, using 10 replications for each *K*, to determine cross‐entropy. The results were visualized using the POPHELPER Structure Web App v1.0.10 (Francis 2017). Finally, we calculated the pairwise F_ST_ between populations based solely on the outlier SNPs, to quantify the degree of genetic differentiation driven by potentially adaptive loci, as higher pairwise F_ST_ values between populations based on these SNPs would indicate stronger divergence due to selection.

## 3 Results

### 3.1 *mt*DNA Variation

We used 13 markers, of which 11 were polymorphic, providing 24 haplotypes in 841 trees in the examined stands of Polish Scots pine populations. Of 24, only 12 main haplotypes that occurred more than three times were used to recreate the haplotype network and illustrate their geographic distribution (Figure 1). The final data set consisted of 810 trees with nine polymorphic sites. Overall, the haplotype network revealed two major groups of haplotypes, one including H1 with closely related H3 and several other less frequent haplotypes, and H2 that, together with a few less frequent ones, consisted of a distant haplogroup. The two most frequent haplotypes (H1, H2) were present in every population and in 70% of individual trees, in general. The third most frequent haplotype (H3) occurred in 15% of the trees, was present in 24 of 27 populations, and prevailing in stands from the north‐east part of Poland. Although H1 and H2 are not very closely related, both occurred together in all populations (Figure 1). Most of the populations studied were characterized by a high haplotypes richness (Table 1). The mean haplotype diversity for all samples was Hd = 0.661; the lowest average haplotype diversity was detected in the Międzylesie population (Hd = 0.301), while the highest average haplotype diversity was detected in Gubin, Bolewice, and Międzyrzec (0.789, 0.766, and 0.755, respectively; Table 1). There was no significant relationship between the measures of genetic diversity and the geographical distribution of the populations. Analysis of genetic relationships between haplotypes using UPGMA revealed a division between two groups as in the case of the haplotype network (Figure S2). The lack of phylogeographic structure was observed in the Mantel test (*r* ^2^ = 0.0012, *p* = 0.350) and the comparisons of N_ST_ and G_ST_ (N_ST_ = 0.0372, G_ST_ = 0.0293) (Figure S3). SAMOVA analysis revealed 3 groups for K3 with low differentiation level. The first two included only two populations from southern Poland (group 1 = KB, group 2 = Md), while the third group consisted of the remaining 25 populations (Figure S4).

**TABLE 1:** Measures of genetic variation at *mt*DNA, *n*SSR and SNPs in the analyzed populations of scots pine.

Population (Acronym, RP*)		*mt*DNA				*n*SSR							SNPs			
	N	H	Ne	Rh	Hd	A	A_r_	A_p_	H_o_	H_e_	F_IS_*	F_IS_**	K	H_o_	H_e_	F_IS_***
Barlinek (B, So30)	30	5	2.961	3.893	0.685	6.750	6.683	0	0.531	0.542	0.073	0.044	10.152	0.327	0.328	0.0012
Bolewice (Bo, So33)	36	6	3.879	4.822	0.766	7.375	6.939	4	0.521	0.540	0.049	0.028	10.104	0.326	0.327	0.0020
Bytów (By, So11)	31	7	3.546	5.484	0.742	6.938	6.825	0	0.575	0.572	0.012	0.016	10.224	0.327	0.327	0.0001
Chełmiec (Ch, So80)	32	4	1.931	2.800	0.499	7.375	7.153	1	0.558	0.558	0.046	0.021	10.345	0.327	0.328	0.0025
Czarne (Cz, So31)	30	6	1.948	4.700	0.503	7.000	6.937	0	0.515	0.541	0.065	0.042	10.247	0.326	0.327	0.0017
Goleniów (Go, So40)	31	6	2.445	4.484	0.611	7.188	7.048	2	0.589	0.570	−0.017	0.006	9799	0.327	0.327	−0.0001
Gubin (G, So34)	32	7	4.206	5.879	0.789	7.438	7.223	1	0.531	0.542	0.035	0.024	9703	0.327	0.327	−0.0013
Hajnówka (H, So23)	30	7	3.556	6.000	0.746	7.063	6.999	1	0.528	0.553	0.062	0.024	10342	0.329	0.328	−0.0015
Janów L. (J, So62)	30	4	2.739	2.931	0.658	7.000	6.933	0	0.535	0.546	0.017	0.013	10.611	0.328	0.327	−0.0025
Kaliska (K, So32)	30	5	2.885	3.800	0.676	6.688	6.629	1	0.535	0.548	0.040	0.041	9957	0.330	0.328	−0.0038
Kobiór (KB, So60)	30	7	2.993	5.724	0.69	6.938	6.877	3	0.546	0.570	0.059	0.014	9950	0.327	0.327	−0.0014
Lipnica (L, So80)	30	4	2.722	2.998	0.655	6.750	6.685	2	0.535	0.546	0.036	0.021	10.233	0.325	0.327	0.0067
Międzylesie (Md, So70)	33	3	1.411	1.993	0.301	6.875	6.643	1	0.549	0.561	0.036	0.022	9787	0.326	0.325	−0.0031
Międzyrzec (Ms, So42)	32	7	3.710	5.843	0.755	7.000	6.872	3	0.498	0.527	0.071	0.028	10.277	0.327	0.327	0.0005
Milicz (M, So30)	32	6	3.130	4.844	0.703	7.063	6.890	1	0.568	0.557	−0.004	0.011	9537	0.328	0.328	−0.0032
Miłomłyn (Mi, So12)	30	5	3.193	3.742	0.71	7.000	6.881	0	0.542	0.544	0.020	0.017	10.728	0.329	0.329	0.0014
Pisz‐Dziadki (Pi, So21)	30	5	2.885	3.800	0.676	7.750	7.654	4	0.553	0.543	−0.001	0.208	10.067	0.328	0.328	0.0010
Płońsk (Pl, So40)	30	6	2.761	4.893	0.661	7.438	7.360	0	0.547	0.549	0.020	0.011	10.455	0.327	0.328	0.0022
Pomorze (Po, So20)	32	7	2.707	5.830	0.652	6.938	6.751	0	0.560	0.534	−0.033	0.006	10.275	0.327	0.327	−0.0010
Prószków (P, So50)	32	5	3.483	3.978	0.736	6.938	6.776	1	0.553	0.557	0.024	0.013	10.018	0.328	0.327	−0.0008
Rychtal (R, So52)	34	5	2.563	3.729	0.63	7.125	6.853	1	0.529	0.534	0.024	0.014	10.464	0.329	0.329	−0.0020
Spała (S, So61)	31	6	2.711	4.693	0.653	7.063	6.914	0	0.548	0.535	−0.008	0.007	10.273	0.327	0.327	−0.0008
Supraśl (Su, So24)	31	5	3.280	3.974	0.718	7.500	7.347	2	0.530	0.545	0.044	0.023	10.390	0.327	0.327	0.0017
Węgliniec (We, So51)	32	6	2.142	4.529	0.55	7.500	7.268	0	0.525	0.541	0.045	0.014	9742	0.327	0.326	−0.0014
Wichrowo (Wi, So20)	30	5	2.651	4.000	0.647	6.938	6.866	1	0.556	0.554	0.012	0.010	10.388	0.328	0.327	−0.0024
Włocławek (Wl, So30)	30	6	2.882	4.893	0.677	7.375	7.302	2	0.513	0.549	0.083	0.032	9614	0.327	0.327	−0.0004
Wyszków (Wy, So41)	30	8	3.571	6.686	0.745	7.188	7.110	0	0.517	0.529	0.040	0.016	10.429	0.327	0.327	0.0006
**Mean**	**31**	**5.7**	**2.922**	**4.479**	**0.661**	**7.118**	**6.978**	**1.1**	**0.540**	**0.548**	**0.033**	**0.018**	**10.152**	**0.327**	**0.327**	**−0.0002**

Abbreviations: A, mean number of alleles; A_r_, allelic richness; Ap, number of private alleles; F_IS_*, fixation index calculated with Fstat; F_IS_**, fixation index calculated with INEST; F_IS_***, inbreeding coefficient averaged over SNPs; H, number of haplotypes; Hd, haplotype diversity; He, expected heterozygosity; Ho, observed heterozygosity; K, average number of nucleotide difference; N, number of samples; Ne, effective number of haplotypes; Rh, haplotypic richness; RP, Regions of provenance; bold values indicate mean of basic statitistic across all populations.

### 3.2 *n*SSR Variation

**FIGURE 2:**
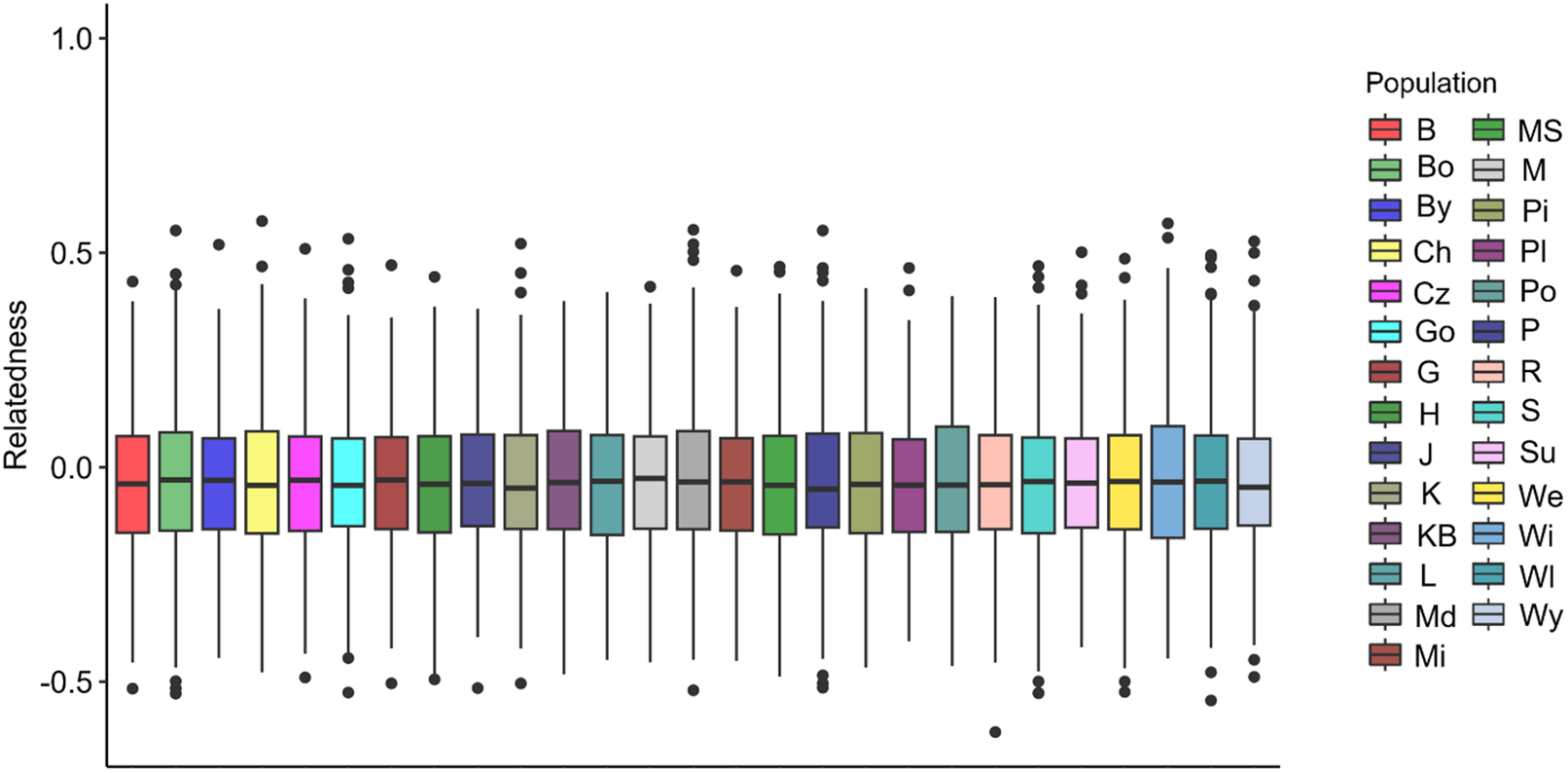
Boxplots showing mean relatedness among individuals across 27 studied pine populations based on all SNPs.

**FIGURE 3:**
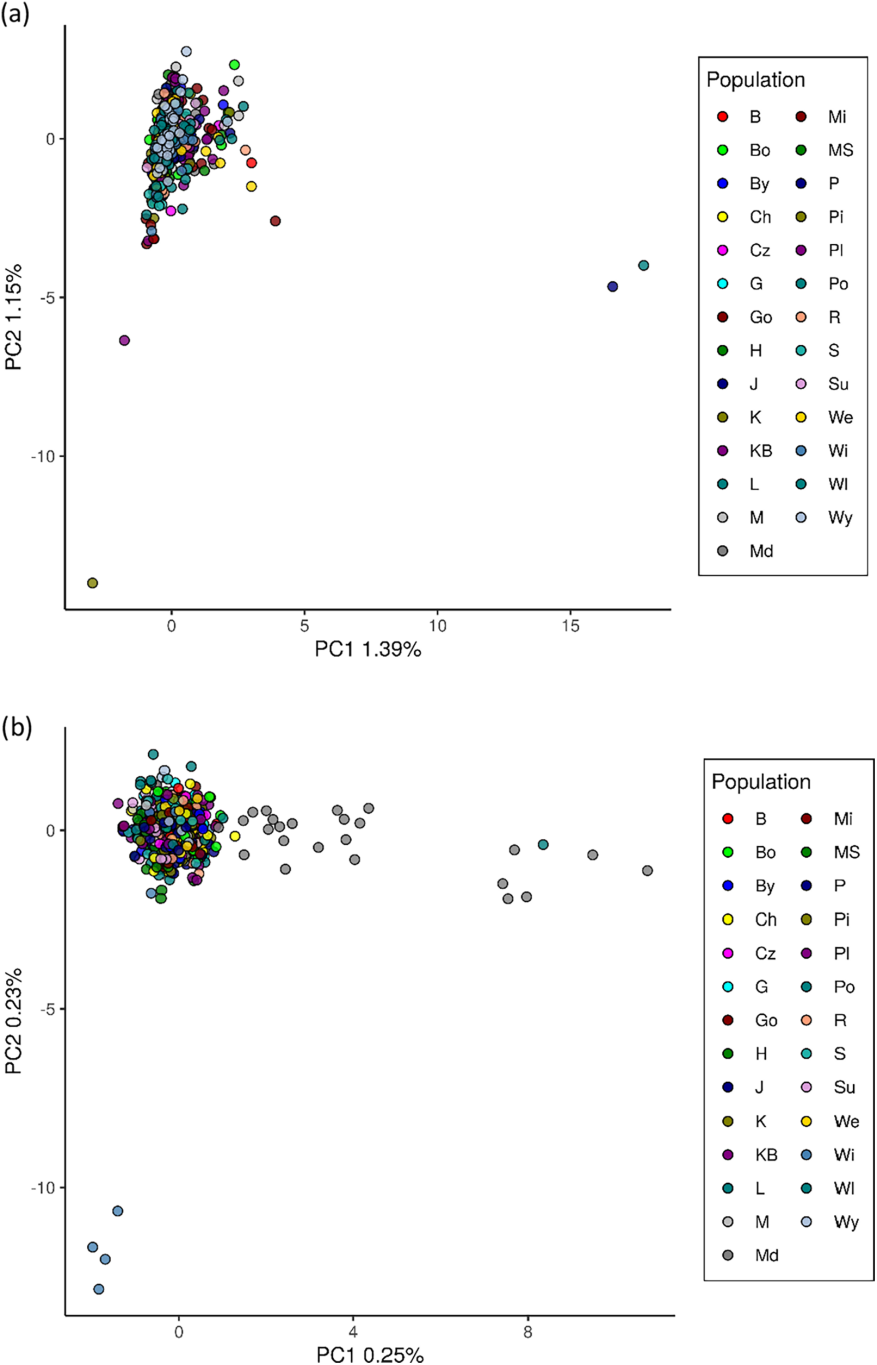
Principal Component Analysis at *n*SSR loci (a) and all SNP loci (b) showing relationships between studied individuals. Most outlier individuals at SNPs loci were from population Md and Wi.

**FIGURE 4:**
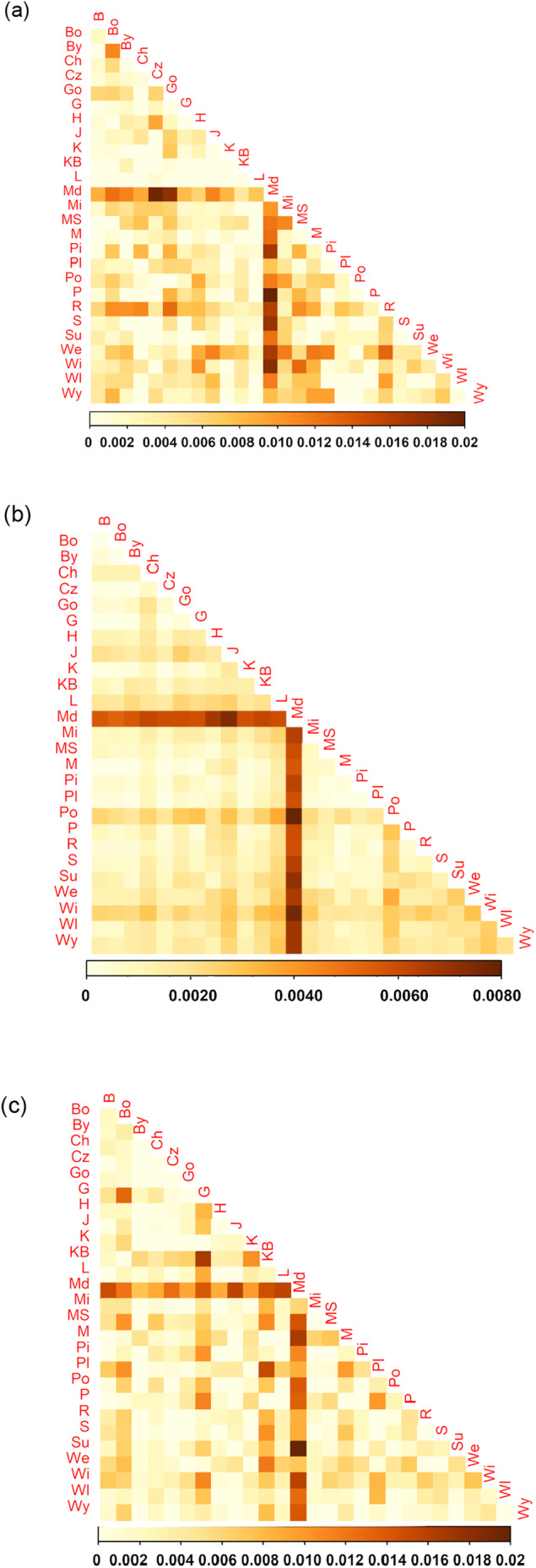
Heat map of F_ST_ values between analyzed populations of Scots pine for *n*SSR (a) SNPs (b) and outlier SNPs (c). Order of populations as in Table 1.

The final data set consisted of 16 polymorphic loci with mean frequency of null alleles in all populations. Null = 0.009 (range, 0.003–0.014) that did not exceed the cut‐off point of Null = 0.19 (Chapuis and Estoup 2006). The highest average number of alleles and alleles richness was observed in Pisz (A = 7.75, A_r_ = 7.65) from the northeastern Poland (provenance region So21) and the lowest in Kaliska (A = 6.69) from northern Poland (So32) and Międzylesie (A_r_ = 6.64) from southwestern Poland (So70), respectively (Table 1). Only a few private alleles were present in some populations. The observed heterozygosity was at a comparable level in all populations and ranged from 0.498 to 0.575, with an average of 0.540 (Table 1). No deviation from HWE equilibrium was found in the populations, and the F_IS_ index was relatively low or negative. Results of relatedness analysis showed that average kinship between individuals within population is substantially low (mean values of Queller‐Goodnight relatedness measure were close to 0), and consistent across all populations. There was no qualitative difference when considering the SSR or SNP dataset and we show result based on SNPs only (Figure 2). No single population showed excess of highly related individuals and thus no individual was removed from further population structure analysis. PCA and PCoA analysis reveal a uniform distribution of genetic variation in Polish Scots pine populations (Figure 3a and Figure S5a). No signatures of the population structure were indicated in Bayesian clustering with STRUCTURE (Figure S6). Genetic differentiation between the populations was generally low; based on the F_ST_ values the most divergent was the population of Międzylesie (Figure 4a). The global F_ST_ conducted in FreeNA was equal to 0.0028 and 0.0023 with and without null alleles, respectively. POPS 1.2 results were fully consistent with STRUCTURE analysis indicating homogeneous genetic structure of examined Scots pine populations.

### 3.3 SNPs Variation

All genotyped samples passed the quality threshold and were used in the analyses. From the initial set of 43,565 genotyped SNPs, 5968 loci were filtered as they exceeded our missing data cutoff level, appeared monomorphic in this sample set, had MAF < 1% or significantly deviated from HWE. The 37,597 resulting markers were considered a “complete” set of SNPs. In this set, 44,688 potential LD pairs were found that involved a total of 26,013 unique loci. After strict pruning of the linked loci, the new set, hereafter “LD pruned,” contained 11,583 markers. Both the “whole” and “LD‐pruned” SNP sets were used in parallel in subsequent analysis. The populations showed very similar patterns of genetic variation at two sets of SNPs, and here we summarize only the results obtained from the whole set. High within‐population genetic diversity and low between‐population genetic variation were observed across populations. No fixed or private SNP alleles were found in any population. Nucleotide differences between samples within populations ranged from 9537 (Milicz) to 10,728 (Miłomłyn), with a mean over stands of 10,152 (Table 1). However, when all samples were treated as a group, this average distance dropped to 1624. In each population, about 98.96% of the loci appeared heterozygous. The observed and expected heterozygosity estimates were very similar, and the population‐specific values hardly deviated from their mean values (0.327 (SD 0.001) the same for both). There was no indication of inbreeding in any population. The average F_IS_ statistic over loci was −0.0002 (Table 1), and the median values of inbreeding statistic across the populations were close to 0 (Figure S7). No significant correlation was found between genetic and geographic distances (*r* ^2^ = −0.161, *p*‐value = 0.927) (Figure S8). Close genetic relationships between populations, with exception of most individuals from Międzylesie (Md, So70) and Wichrowo (Wi, So20) were observed in the PCA and PCoA analyses (Figure 3b and Figure S9). In general, genetic differentiation between Scots pine populations was found to be very low, F_ST_ = 0.0017 (Figure 4b). The most differentiated pair of populations were Międzylesie and Pomorze sites (F_ST_ = 0.0075); however, the LD‐pruned set indicated Międzylesie and Wichrowo populations as the least similar in terms of allele frequencies (F_ST_ = 0.0080). The low differentiation observed between populations was confirmed by AMOVA, which showed that the gross variation (more than 99%) observed in the Polish population of Scots pine segregates within individual genomes (Table S2). Along with this, the only significant fixation index in the analysis was the inbreeding coefficient of an individual relative to the total population—F_IT_ (Table S3). STRUCTURE analysis showed that the populations defined here based on the sampling locations are all mixtures of many different genetic clusters, and that there was a large variation between runs in the way the samples were assigned. In general, no clear clustering of samples was identified in the “admixture” as well as the “no admixture” version of the STRUCTURE algorithm. No pattern was observed in the summary statistics used for the selection of the best K; they differed weakly between successive K values and were not “phased” between subsets (Figure S10). In terms of genetic distinction, none of the sampling populations exists as a discrete unit, and no other structure was identified.

Using our complete dataset of 37,957 SNPs, we identified a total of 96 outlier SNPs using the pcadapt method and 4 outlier SNPs using OutFLANK (Figure S11). In particular, the number of outlier SNPs detected by pcadapt varied with the FDR threshold: at a more stringent *q*‐value of 0.05, only 48 SNPs were identified as outliers. However, the number of outliers detected by OutFLANK remained consistent regardless of the FDR threshold. Surprisingly, only a single SNP was identified as an outlier by both pcadapt and OutFLANK, highlighting the distinct sensitivities and specificities of these two methods. All the 99 outlier SNPs constituted 0.26% of the total SNP dataset, indicating a minimal overall impact on population structure. This was further corroborated by PCA, where the inclusion or exclusion of these outliers had only a slight effect on the overall pattern observed. Specifically, when outliers were excluded, there was a modest increase in variance explained by the first principal component (PC1), from 0.27% to 2.03%. Despite this, most of the individuals of all populations still clustered closely in the PCA, forming a central group. The only notable deviation was the consistent positioning of individuals in the Md population, which remained distinct regardless of whether outliers were included in the analysis (Figure S9). However, the explained variance was significantly higher when looking at PCA based only on outlier SNPs (PC1 = 16.09%, PC2 = 7.16%), and there was split into three groups: individuals from different populations were mixed between these groups without a clear population‐specific or regions of provenance‐specific pattern. This suggests that outlier SNPs capture little variation that differentiates individuals. However, those individuals do not cluster to the particular predefined population or regions of provenance categories. This was also evident when considering the results of the LEA analysis, where the cross entropy decreased sharply at *K* = 1 and reached its minimum at *K* = 15. These findings suggest that outlier SNPs do not differentiate effectively between populations, since when *K* = 15 no distinct population‐specific clusters were observed; instead, individuals of various ancestry were present in all populations, albeit in differing proportions. Differentiation between populations based on outlier SNP pairwise F_ST_ was an order of magnitude higher than when all SNPs were used, but still quite weak, with maximum less than 2% differentiation (Figure S12). The F_ST_ values obtained from the SSR markers were comparable to those derived from the SNP outliers, both showing a maximum F_ST_ of 0.02 and producing similar heatmaps (Figure 4c).

## 4 Discussion

### 4.1 Genetic Relationships Between Populations

In this study, we genotyped and analyzed the distribution of polymorphisms in thousands of genetic markers in a large panel of more than 800 individuals derived from 27 of the most valuable phenotypically and ecologically diverged populations of Scots pine in Poland. Genetic variation based on molecular markers derived from genomes of different modes of transmission and inheritance in pines was compared between populations representing different stands and all regions of provenance defined for the species in the country. Proper management of the genetic resources of Scots pine, which is one of the most economically important species for timber production in many European countries, is challenging given the ongoing climate changes and the increased mortality in the range of species. In general, Scots pine populations from Central Europe, including the area analyzed in Poland, demonstrated fast growth rate, high wood quality, fitness, and breeding value on many provenance trails conducted both locally in Poland and also in Eurasia and North America, for example (Barzdajn, Kowalkowski, and Chmura 2016; Giertych and Oleksyn 1992; Stephan and Liesebach 1996). Specifically, the results indicated a large variation in the growth characteristics of Scots pine at the level of a given provenance (Giertych 1993; Przybylski, Matras, and Sułkowska 2015; Szeligowski et al. 2016; Oleksyn and Rachwał 1994).

Until now, most of the genetic studies of Scots pine in Poland refer to the application of a limited number of stands and genetic markers (Wójkiewicz, Litkowiec, and Wachowiak 2016; Be 2016; Wachowiak et al. 2014). However, the sampling and resolution of the markers were usually too low to draw any general conclusions about the genetic relationships between native ecotypes. Furthermore, the regions of provenance of the species were never fully represented in population genetics and genomic studies. Our data show striking genetic similarity at background genetic variation between populations suggesting their common population history. Taking into account their age, they were initiated at the time when different parts of the analyzed territory were, due to the historical geopolitical situation, under the Austro‐Hungarian, Prussian, and Russian administrations. Active forest management was in operation at that time, with varying intensity among these different administrative regions. It cannot be excluded that some Scots pine seeds were possibly traded, but perhaps at much smaller scale compared to Norway spruce, due to high availability of local seed sources (Jansen, Konrad, and Geburek 2017; Wachowiak et al. 2024). Therefore, it seems unlikely that analyzed pine populations were established as a result of historical seed transfer from some common sources. Considering the high genetic similarity among the populations studied, they most likely represent natural stands in the area that was recolonized over the last several thousand years.

Mitochondrial DNA, due to the limited ability of seed transfer, allows one to identify fine‐scale differentiation between populations compared to pollen‐mediated genomes. Gene flow by pollen has a homogenizing effect on population structure and is strong enough to obscure ancestral history in nuclear loci (Bruxaux et al. 2024). Mitochondrial DNA analysis indicated that two main mitotypes (H1 and H2) belong to distinct genetic groups that were fixed in 70% of all individuals. Haplotype H1 corresponds to the main European mitotype present in most southern and western populations, while H2 corresponds to the main Fennoscandian haplotype that is abundant in Finnish stands (Wachowiak et al. 2023). The third most common mitotype, H3, was present in 24 of the 27 populations studied and 15% of all trees analyzed and was dominated by populations from north‐eastern Poland. The remaining haplotypes were much less frequent. In general, most of the populations studied were characterized by high haplotype richness, and the results coincide with previous reports showing the highest *mt*DNA in mid‐latitude regions (Wachowiak et al. 2023). The results suggest that during the postglacial recolonization process populations of different origins admixed in the central European distribution of the species and newly established Scots pine forests did not diverge significantly, resulting in populations that share a common history. The hypothesis of admixture for the origin of Polish populations is further supported by the results of genetic variation at neutral loci, including SSR and SNPs data. Here, all sampled populations were classified as a single genetic group in STRUCTURE and POPS analyses and showed a similar level of genetic variation. Comparable genetic relationships between most samples were also observed in the PCA analysis. The level of differentiation between populations at the SSR loci (average F_ST_ = 0.002) and SNP markers (F_ST_ = 0.002) was marginal and lower compared to some earlier estimates in the populations of Polish Scots pine at the SSR loci (F_ST_ = ~0.03, Hebda and Wachowiak 2019). The most recent study based on a wide range of Scots pines analyzed on nuclear SNP markers revealed relatively low variation between populations (F_ST_ = 0.048, Bruxaux et al. 2024). In our study, outlier SNPs did not show any signatures of population differentiation that would correspond to the predefined regions of provenance. Other studies show that there is little correlation between local adaptation and genetic data (Hall et al. 2021; Tyrmi et al. 2020) which does not align with the high morphological diversity observed due to possibly non‐genetic origin of some traits variation and adaptation linked to many genes with small individual effects. Certainly, the populations included in previous Scots pine studies did not refer to the oldest stands of the species or were established from open pollinated seeds from those stands, potentially affected by gene flow from much younger commercial stands of unknown origin. Collectively, the results indicate a very uniform genetic background of the most valuable Polish Scots pine populations that were most likely established through gene exchange in large geographical areas that led to high genetic diversity within individual stands and no significant differentiation between populations.

Due to the effectively non‐existing population structure in our dataset, a similar pattern of population differentiation observed at SSR and SNP markers does not reflect a higher resolution of the SNP array in genetic diversity and fine‐scale population studies (Zimmerman, Aldridge, and Oyler‐McCance 2020). In our recent investigations, the SNP array showed much better performance in delineating populations and hybrids of closely related pine species compared to SSR markers (Łabiszak et al. in preparation). In general, both mitochondrial markers and nuclear DNA SNPs are useful for population delineation. The first set of markers that is transmitted maternally without sexual recombination can mark distinct genetic linages and therefore are suitable for phylogeographic studies, while the nuclear markers can additionally indicate targets of natural selection. Our results support the hypothesis of a common origin of the populations and do not provide an indication of a population structure in the studied area, in contrast to that recently described for the range of other parts of the species distribution (Łabiszak and Wachowiak 2023). More recent studies indicated several genetic linages of species that intermixed at some parts of the species range during recolonization but maintained their distinct genetic characteristics despite presumably intensive gene flow between populations over large geographical areas (Łabiszak and Wachowiak 2023; Dering et al. 2017). Significant divergence between populations was also observed in southern refugial regions (Pyhäjärvi, Salmela, and Savolainen 2008; Dering et al. 2021). In wind‐pollinated tree species with possible long‐distance pollen dispersion events, without strong geographical barriers at regional scale and quite uniform environmental conditions, we should expect a shallow genetic structure and similar genetic background. However, such a fine‐scale structure could possibly be maintained if there are some phenological differences between populations, or selection against maladapted alleles would affect population fitness. Due to many shared haplotypes between populations, only two stands from southern Poland (Międzylesie, Kobiór) were indicated as genetically distinct in the SAMOVA analysis. Furthermore, the population of Międzylesie (Md, So70) in the Kłodzko Valley surrounded by the mountains was characterized by the lowest number of mitotypes and mitotype diversity, the low number of SSR alleles and their diversity, as well as one of the lowest estimates of heterozygosity based on SNPs, and was consistently found to be the outlier at the nuclear loci in the PCA and PCoA analyses. Although the non‐native origin of that population cannot be excluded, the geographic region where it is located was previously shown to have unique genetic characteristics in other tree species (e.g., *Pinus mugo* (Żukowska and Wachowiak 2017)) and recent evidence indicates the possible presence of a small isolated glacial refugium nearby, which could ensure the persistence of some genetic preglacial remnants in the region (Suchan, Malicki, and Ronikier 2019).

### 4.2 Forest Management Implications

Our results have important implications for forest management, as they contribute to the discussion of the boundaries of the provenance regions and the transfers of FRM. The populations analyzed in the study were derived from the 24 regions of provenance defined for Scots pine in Poland that are managed under strict seed transfer regimes. Consequently, the reproductive material derived from a given seed stand should be planted within a given region of provenance, and there are restrictions on the movement of the plant material between the regions. These rules are especially strict for some regions of provenance, which are considered sources of the most valuable populations of Scots pine in Poland. Similar rules were also in operation in previous seed regionalization regulations, where most of these regions were considered “maternal” versus the others, which were considered ordinary regions (Załęski et al. 1994). However, no formal tests were conducted to compare the silvicultural properties of the reproductive material derived from these two types of regions. Some provenance tests in which such a comparison is possible do not prove the superiority of one versus another in terms of individual tree growth and stand productivity (Chmura, Guzicka, and Rożkowski 2021). Furthermore, in these tests, local populations are often among the best for tree diameter or height, but usually not for area‐based productivity (Chmura, Guzicka, and Rożkowski 2021; Hebda, Skrzyszewski, and Wachowiak 2017; Hebda, Wachowiak, and Skrzyszewski 2017). However, to date, there has been no coherent comparative assessment of the silvicultural properties of local populations representing different provenance regions. Therefore, although the current delimitation of the provenance regions for Scots pine in Poland is less restrictive than the previous one (Załęski et al. 2000), the ongoing effort to formally test the progeny of seed stands (Sabor et al. 2004) should provide information on whether the diversity in adaptation and quantitative traits still supports this distinction of provenance regions. Such quantitative genetic assessments and progeny tests of the provenance regions combined with environmental and climatic data of the sites would be needed to validate whether the phenotypic differences between the provenance regions are due to local adaptive divergence or plastic responses. Once validated, the application of genomic data related to population history assessments and their signature of adaptation should facilitate decisions on delimitation of provenance regions and support further expansion of its current boundaries into larger areas. Additionally, the condition of selected populations and the viability of seeds from the most valuable provenance regions should be systematically monitored to detect early‐stage symptoms of their environmental stress. The results of genetic differentiation at the molecular level provided by our study do not appear to justify strict rules for seed transfer. Moreover, these rules may be counterproductive in terms of assisted migration in the face of ongoing climate changes (Aitken and Bemmels 2016). It seems reasonable that periodic shortages of reproductive material in a given area could be supplemented with FRM from regions of provenance that match their climatic envelope. Such an approach may be inevitable in the near future, as ongoing and projected environmental changes will significantly affect the fitness and productivity of seed stands, influence suitable climatic niches of forest tree species, and their geographical distributions are likely to follow (Dyderski et al. 2018; Chakraborty et al. 2021). The stress associated with droughts, forest fires, catastrophic winds, insect infestations, and mistletoe outbreaks that are already observed in many European forests, including Scots pine (Taeger et al. 2013), will likely intensify in the future, affecting pine populations. For species with a long generation time, such as Scots pine, the adaptive capacity of their populations is based on standing genetic variation inherited over generations rather than spontaneously arising new mutations (Barrett and Schluter 2008). Therefore, adaptive responses of populations in the near future will depend on the already available genetic variation segregating in the populations. A high level of genetic diversity is crucial to the survival of species because it determines their performance, protects against the detrimental effects of inbreeding, and improves adaptive evolutionary potential and phenotypic plasticity (Alberto et al. 2013). Our data show not only the uniform genetic background of Polish Scots pine, but also indicate that the vast majority (> 99%) of its high genetic diversity is present within each population studied. This pattern is likely related to the common history of populations and is maintained by highly efficient pollen and seed dispersal in this random‐mating and wind‐pollinated species, and its generally large effective population size characterizing forest tree populations (Petit and Hampe 2006). Other genomic studies supported an expansion of current provenance regions of Belgian populations of black alder ( *Alnus glutinosa* ) into larger seed zones (De Kort et al. 2014). However, in our dataset, the most distinct Md population represents southern stands and pines from the mountain regions of Poland are known to be genetically distinct as compared to lowland stands (Wachowiak et al. 2024). Therefore, we recommend treating this population as a distinct provenance region (So70) and FRM should not be mixed with lowland stands.

The climate niche scenarios for the future distribution of Scots pine (Dyderski et al. 2018; Chakraborty et al. 2021) are particularly unfavorable for forests in Poland, where Scots pine is the main tree species. Increased environmental stress will likely broaden the genomic offset between the current genomic composition and the one needed to cope with novel environmental conditions. This will likely negatively affect the fitness of individual trees and whole populations, significantly affecting forest production. Taking this into account, it seems that the most valuable Scots pine in Europe is exposed to increasing environmental stress, occurring at a pace too fast to be followed by local adaptation (Aitken et al. 2008). Taking into account the changing environments, soon the local ecotypes of the species may not be suitable any more at the place of their occurrence. If these scenarios are confirmed, the present‐day regionalization of basic forest material may not hold in the near future. Together, considering existing genomic resources and the uniform distribution of standing genetic variation between populations, our data contribute to the discussion of the revision of existing provenance regions of the species. Similar investigations should also be carried out on other species and in other countries considering that provenance regions are delimited for many forest tree species subject to production and marketing of FRM.

## 5 Conclusions

The research took advantage of the latest genomic resources and developments in Scots pine genomic studies to assess the level and distribution of genetic variation in mitochondrial and nuclear genomes in the most valuable populations of the species in Poland. The study provides new knowledge on the existing genomic resources and the genetic relationships of the ecotypes of the species. The data indicate high genetic similarity between the analyzed populations, suggesting their common history resulting from postglacial recolonization and admixture of populations of different origins. Considering the uniform genetic background of populations and the increasing pressure of environmental changes that globally have a negative impact on the condition and survival of forest tree ecosystems, it appears that the current delineation of provenance regions of Scots pine may need to be revised. Our results do not justify either strict seed transfer rules. We suggest a formal test of the progeny of seed stands to check whether diversity in adaptation and quantitative traits still supports the distinction of particular provenance regions. In parallel, the condition of selected populations including their health status, symptoms of environmental stress (e.g., drought or pest damage), flowering, fructification, and the viability of seeds from the most valuable provenance regions should be systematically monitored to detect early stage symptoms of their environmental stress. It seems reasonable that periodic shortages of reproductive material in a given area could be supplemented with FRM from provenance regions that match their climatic envelope. Together, we believe that this case study provides useful data in the discussion of existing breeding and forest management strategies in Scots pine to counteract the negative consequences of environmental changes. This is consistent with the ongoing efforts and long‐term strategy of the Polish State Forests. Taking into account the similar regionalization practice of FRM in many European countries, the research addresses questions of forest management of general importance in forestry.

## Conflicts of Interest

The authors declare no conflicts of interest.

## Supporting information


Supporting Information S1.

## Data Availability

All genomic data used in this manuscript are available at the Dryad Digital repository: https://doi.org/10.5061/dryad.0cfxpnw9q.
