# Supplementary material for: Genomic Data Support the Revision of Provenance Regions Delimitation for Scots Pine
Source: Evol Appl. 2024 Nov 15;17(11):e70038. doi: 10.1111/eva.70038 (PMC11568063; doi:10.1111/eva.70038)
Supplement: Supplementary file 1 — Supporting Information S1. [file EVA-17-e70038-s001.pdf]

Supplementary Material

Suppl. Table S1. Characteristics of the analysed populations of Scots pine included in the study

Population	Acronym	Region of provenance	Age	Latitude	Longitude	Elevation (m a.s.l)
Barlinek	B	So30	188	52.93	15.181	101
Bolewice	Bo	So33	150	52.375	16.13	103
Bytów	By	So11	146	54.224	17.29	121
Chełmiec	Ch	So80	125	49.642	20.589	532
Czarne_Czl.	Cz	So31	154	53.582	16.986	146
Goleniów	Go	So10	176	53.65	14.695	18
Gubin	G	So34	199	51.981	14.863	71
Hajnówka	H	So23	171	52.619	23.603	180
Janów_Lub.	J	So62	124	50.709	22.31	224
Kaliska	K	So32	215	53.833	18.293	125
Kobiór	KB	So60	186	50.032	18.95	273
Lipnica	L	So80	140	49.722	20.87	503
Międzylesie	Md	So70	171	50.287	16.742	547
Międzyrzec	MS	So42	144	51.965	22.865	164
Milicz	M	So30	155	51.458	17.239	198
Miłomłyn	Mi	So12	162	53.828	19.934	147
Pisz	Pi	So21	158	53.519	21.679	136
Płońsk	Pl	So40	110	52.701	20.551	131
Pomorze	Po	So20	151	53.983	23.451	150
Prószków	P	So50	188	50.559	17.804	208
Rychtal	R	So52	166	51.185	17.957	217
Spała	S	So61	204	51.568	20.203	201
Supraśl	Su	So24	155	53.232	23.362	176
Węgliniec	We	So51	188	51.285	15.2	201
Wichrowo	Wi	So20	182	54.03	20.43	110
Włocławek	Wl	So30	188	52.602	19.119	69
Wyszków	Wy	So41	184	52.661	21.467	129

Suppl. Table S2. AMOVA analysis based on genetic variation among 27 populations of Scots pine

Set		Source of variation	Sum of squares	Variance components	Percentage variation
27 populations	Whole	**Among populations**	175091.4	10.36	0.169
		**Among individuals within populations**	4792847.5	-1.19	-0.019
		**Within individuals**	4959829	6114.14	99.850
		**Total**	9927767.9	6123.31	100
	LD-pruned	**Among populations**	44759.3	2.57	0.164
		**Among individuals within populations**	1228928.9	-0.17	-0.011
		**Within individuals**	1271531	1567.29	99.85
		**Total**	2545219.1	1569.69	100

Suppl. Table S3. Fixation indices and significance test results from AMOVA analysis, showing amounts of heterozygosity at various levels of population structure

SNP set	Fixation index	Value	*p*-value
**Whole**	**FIS**	-0.00020	0.809
	**FST**	0.00169	1.000
	**FIT**	0.00150	0.000
**LD-pruned**	**FIS**	-0.00011	0.608
	**FST**	0.00164	1.000
	**FIT**	0.00153	0.000

Suppl. Figure S1. Regions of provenance for Scots pine in Poland (source: Journal of Laws of September 21, 2015, item 1425. Regulation of the Polish Minister of the Environment of July 29, 2015 on the list, areas and maps of regions of origin of forest reproductive material)

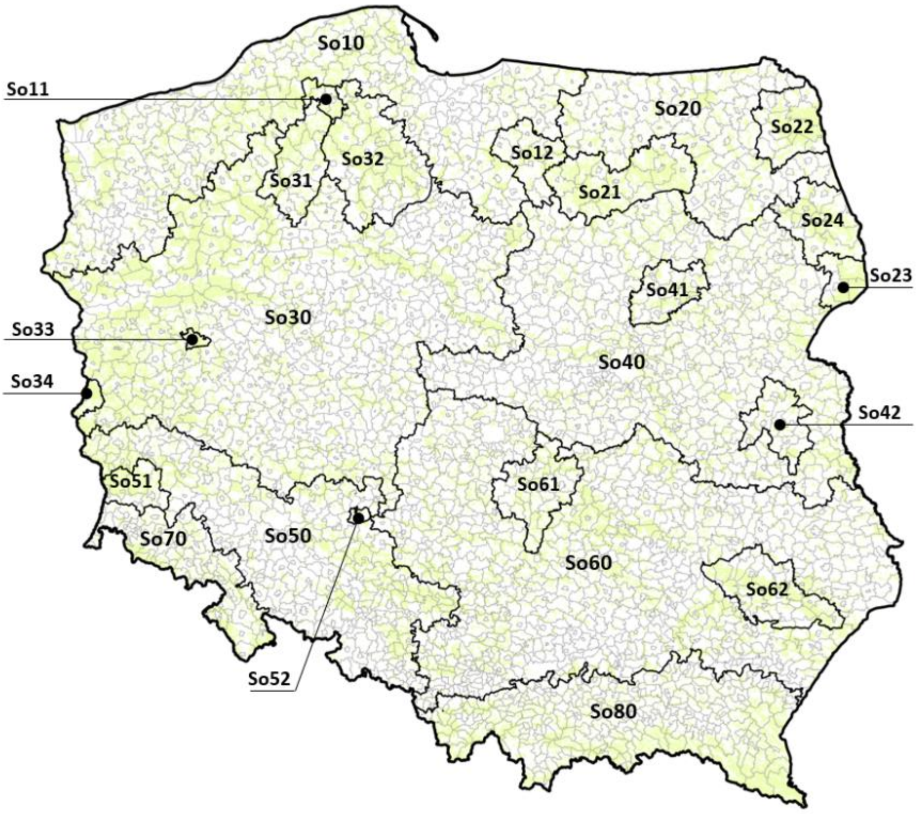

Suppl. Figure S2. UPGMA tree for 12 main haplotypes occurring in 27 Scots pine populations

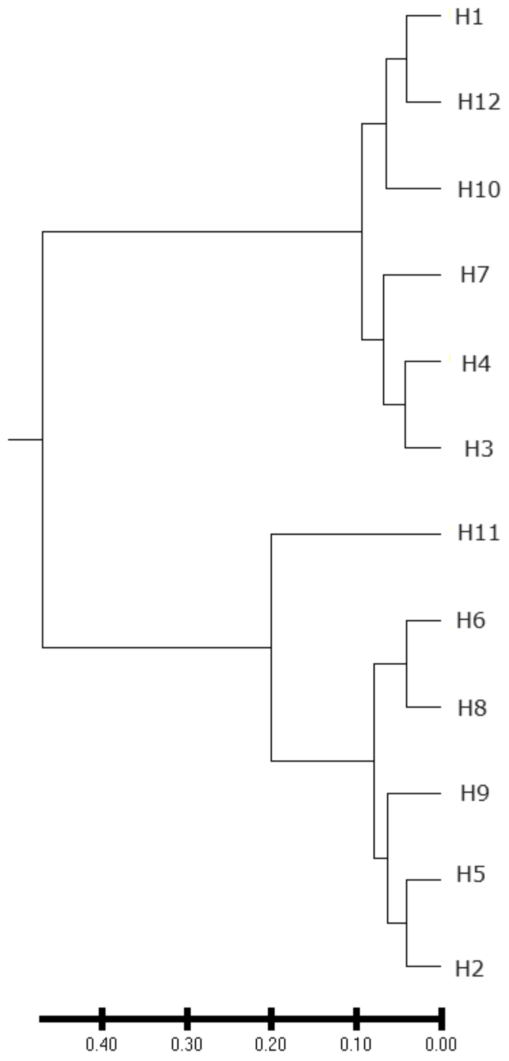

Suppl. Figure S3. Mantel test based on the matrix of genetic distance at *mt*DNA markers and geographical distances between populations

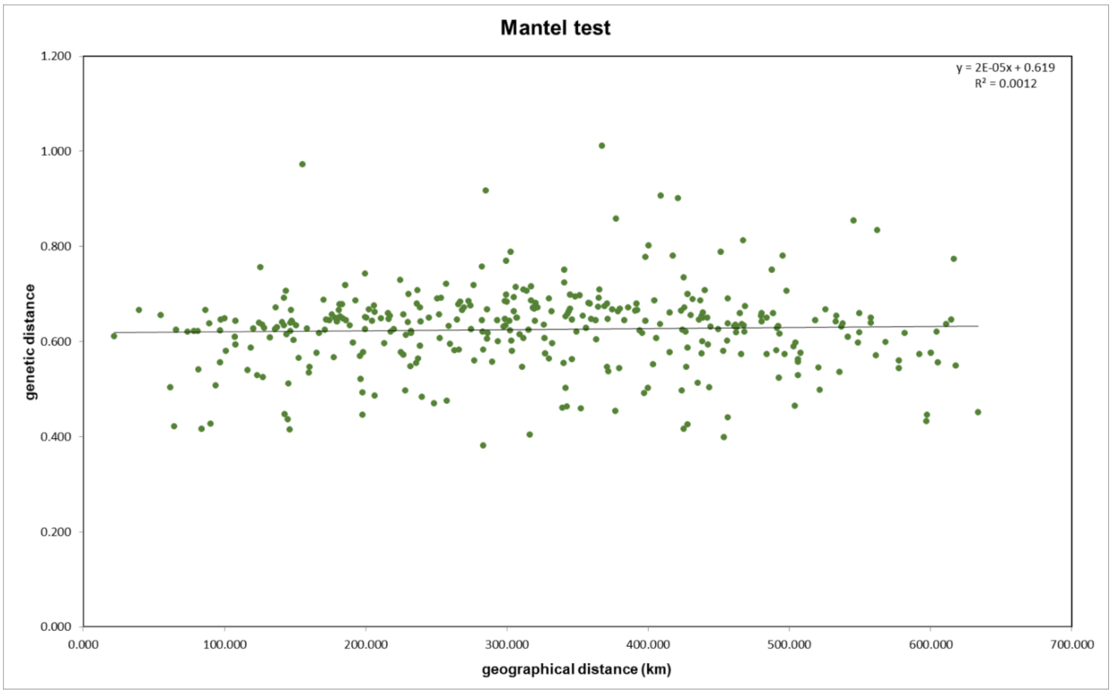

Suppl. Figure S4. SAMOVA analysis for K3 ravels 3 genetic groups. Group1 = "KB", Group2 ="Md", Group3 = 25 remaining populations

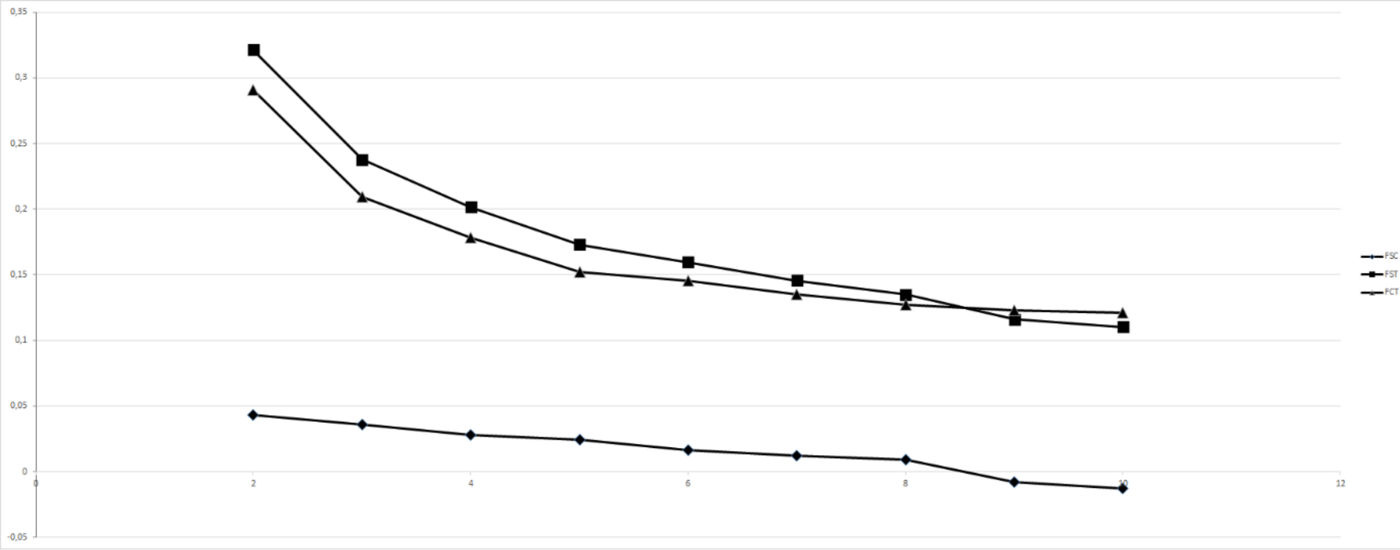

Suppl. Figure S5. Principal Coordinate Analysis (PCoA) showing relationships between studied populations at (a) SSR loci and (b) SNPs markers

(a)

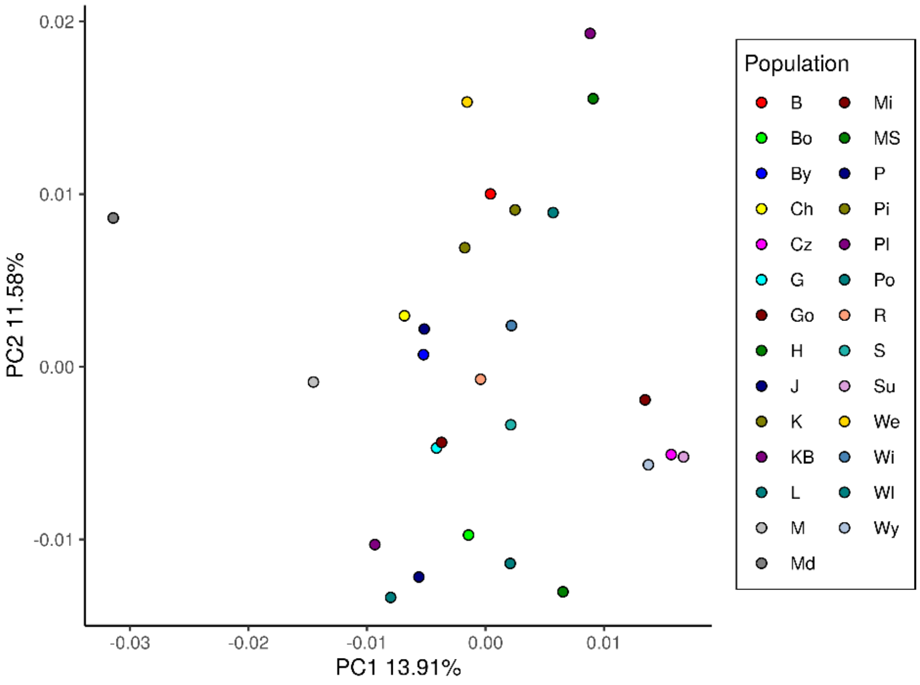

(b)

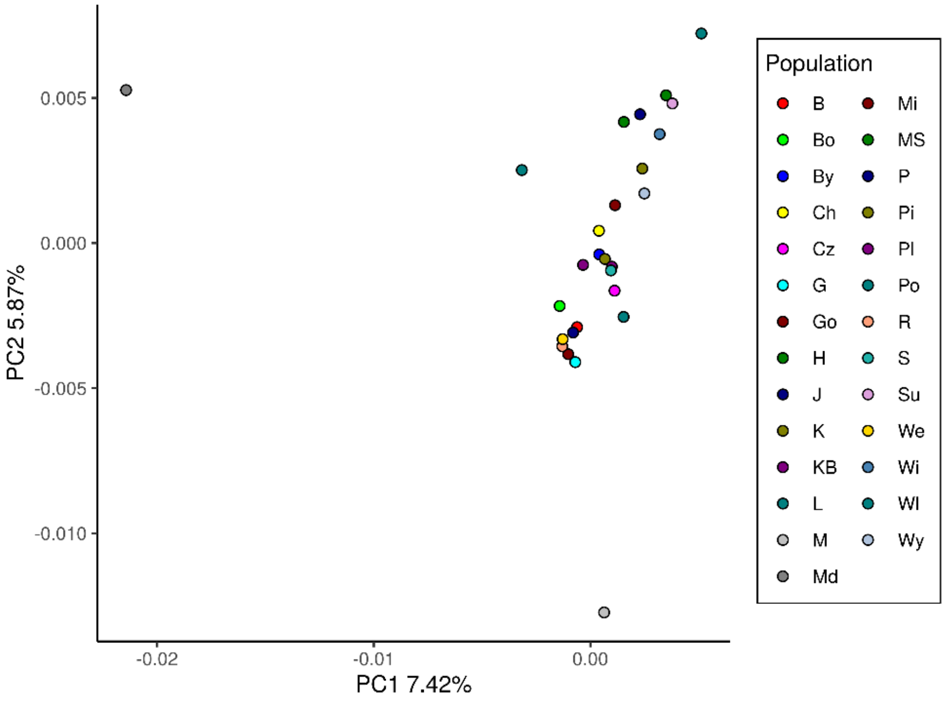

Suppl. Figure S6. Population structure analysis performed using STRUCTURE software

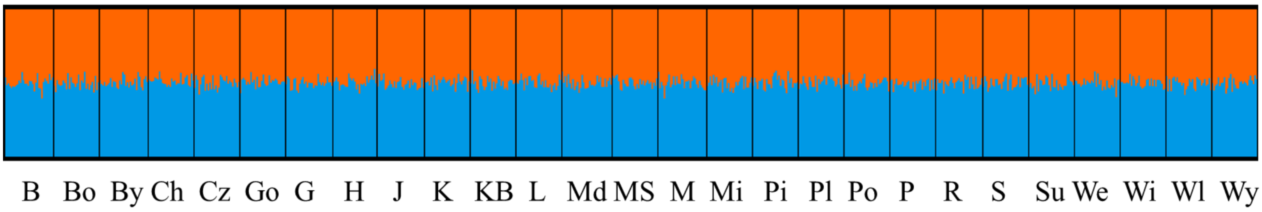

Suppl. Figure S7. Boxplots showing median values of inbreeding across the 27 studied pine populations based on SSR loci

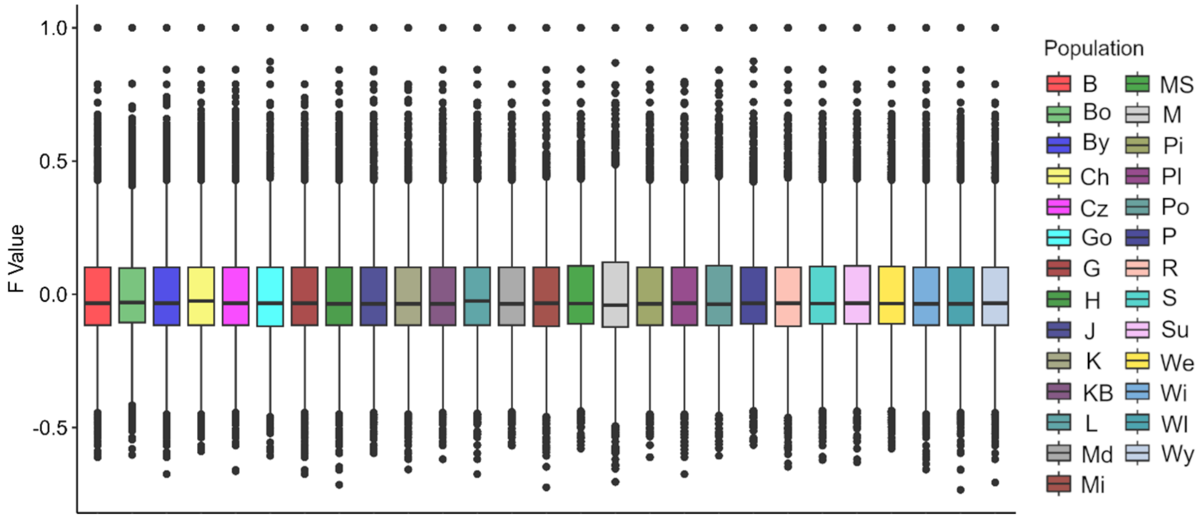

Suppl. Figure S8. Mantel test at SNP markers for the genetic (Edwards distances) and geographic distances between populations (r^2 = -0.161, *p*-value = 0.927)

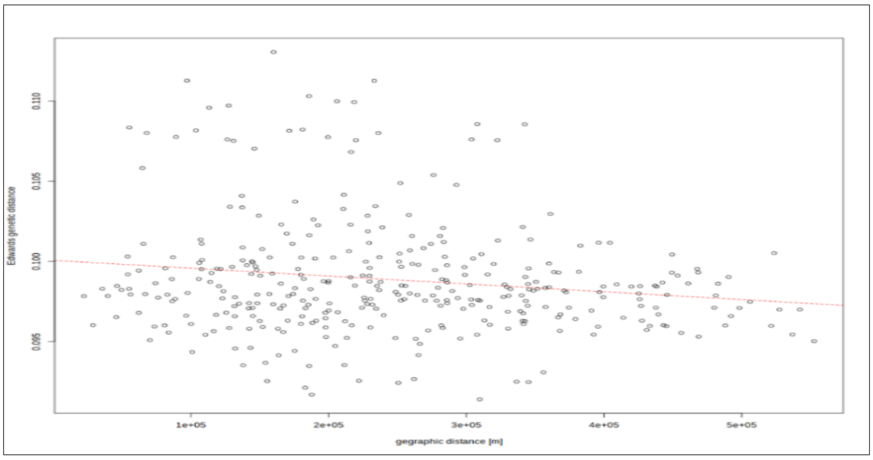

Suppl. Figure S9. Principal Component Analysis (PCA) based on three sets of SNPs: the full set of 37 957 SNPs (a), the set excluding outliers SNPs (b), and the set including only outliers (c)

(a)

(b)

(c)

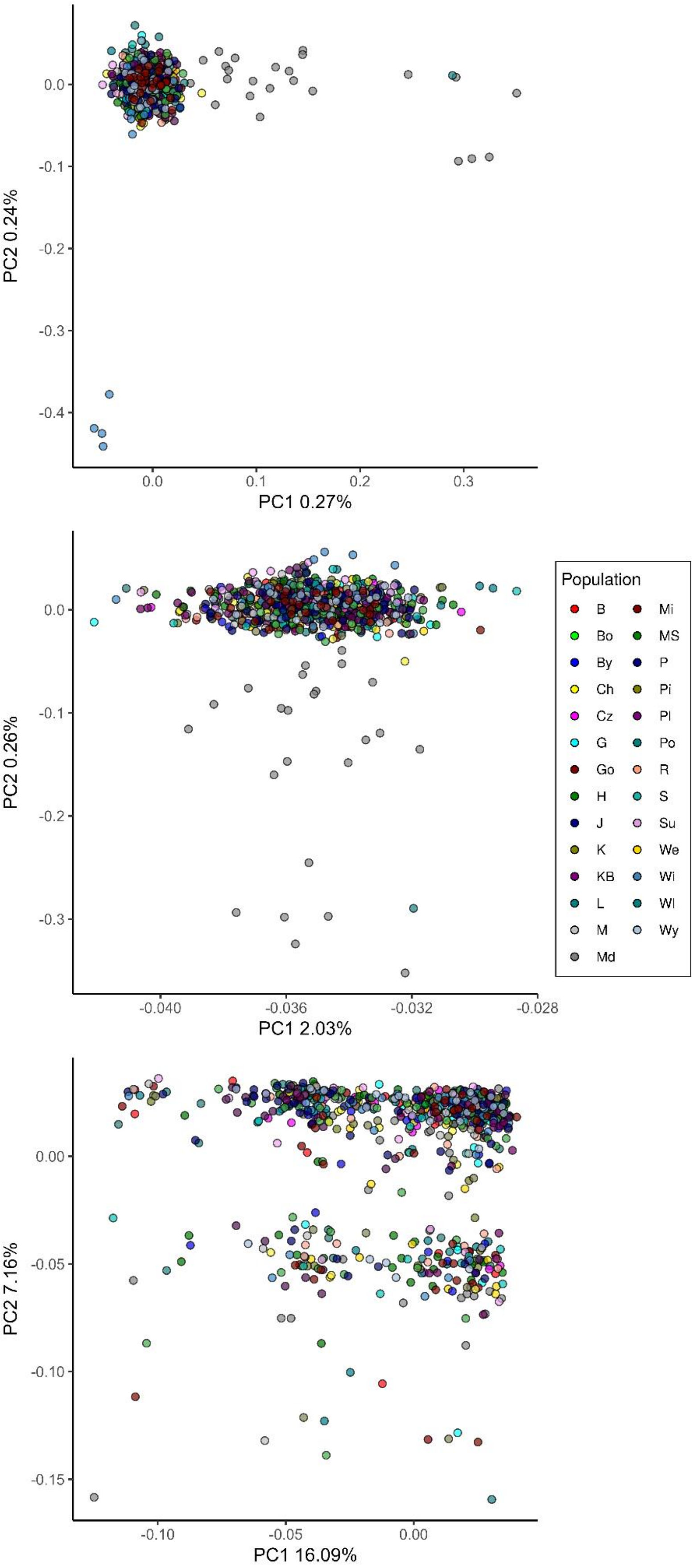

Suppl. Figure S10. Structure analysis (no admixture) results gained by testing 4 subsets of 5,000 random loci from whole SNP set – each represented by different colour. Respective subplots represent main statistics on which inference regarding population genetic structure might be based: lnP(K) mean over 4 replicates; ln’(K); Delta K

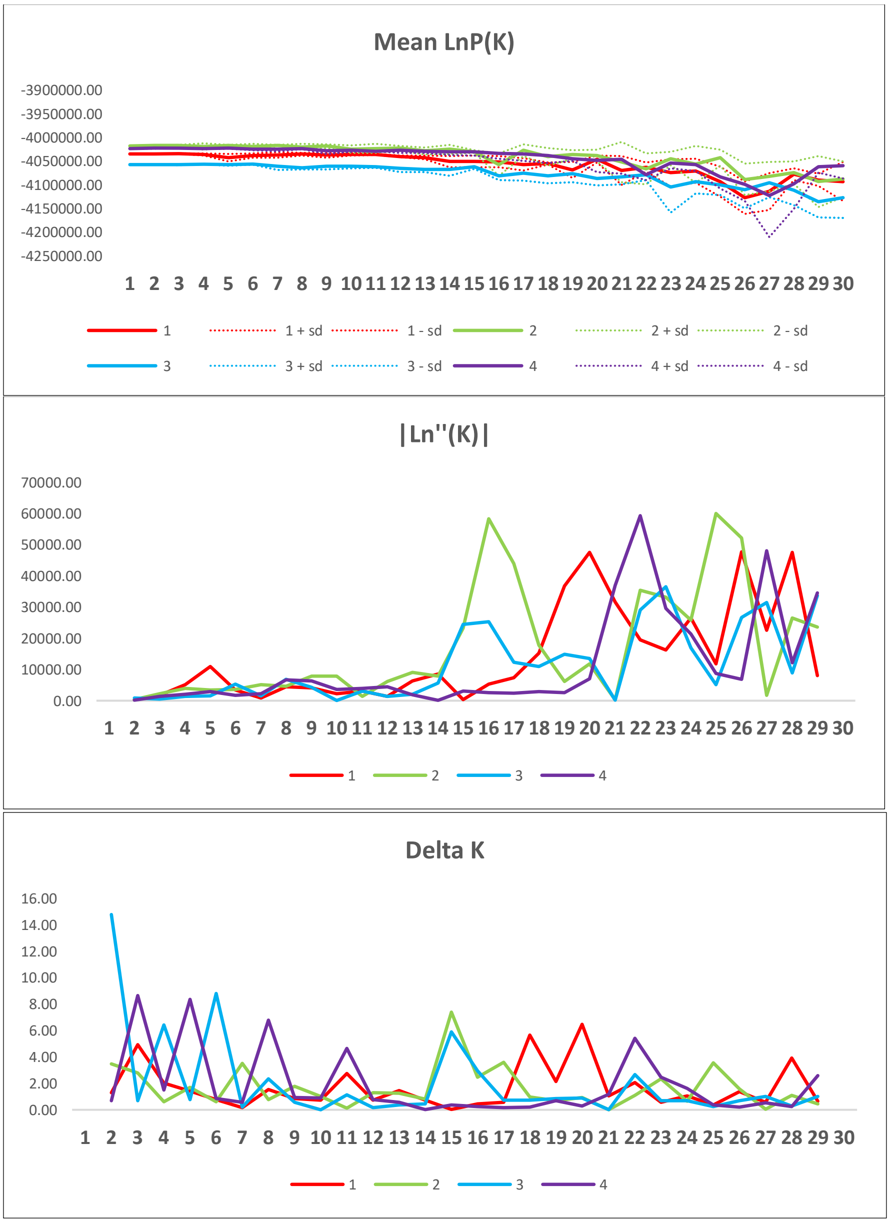

Suppl. Figure S11. Manhattan plots showing the outliers SNPs indicated by pcadapt (a) and OutFLANK (b). Red points denote significant outliers SNPs found at both q-value thresholds. The dashed lines indicate the q-value thresholds (grey = 0.1 and red 0.05, respectively)

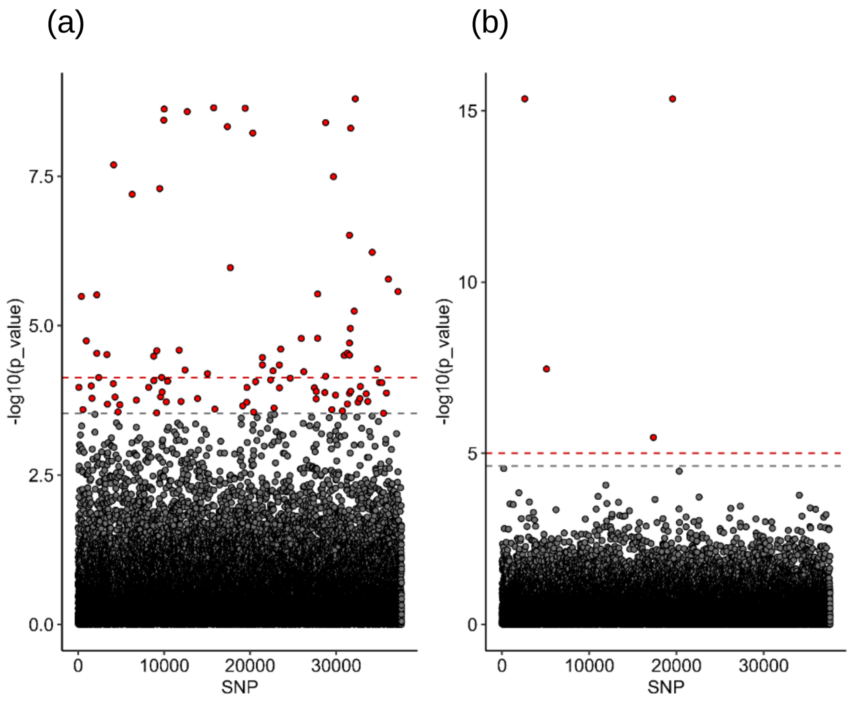

Suppl. Figure S12. Cross entropy between ten different runs for each K in LEA plotted vs. number of ancestral populations. The optimal number of clusters is detected by the first significant drop of cross entropy at K = 1

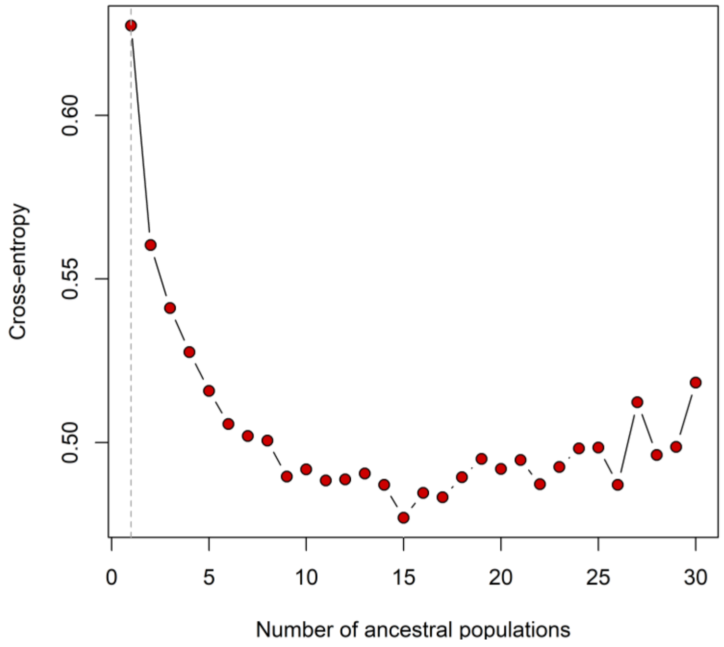

.
